# Correction: SARS-CoV-2 seroepidemiology in Cape Town, South Africa, and implications for future outbreaks in low-income communities

**DOI:** 10.1371/journal.pgph.0004804

**Published:** 2025-06-12

**Authors:** Hannah Hussey, Helena Vreede, Mary-Ann Davies, Alexa Heekes, Emma Kalk, Diana Hardie, Gert van Zyl, Michelle Naidoo, Erna Morden, Jamy-Lee Bam, Nesbert Zinyakatira, Chad M. Centner, Jean Maritz, Jessica Opie, Zivanai Chapanduka, Hassan Mahomed, Mariette Smith, Annibale Cois, David Pienaar, Andrew D. Redd, Wolfgang Preiser, Robert Wilkinson, Andrew Boulle, Nei-yuan Hsiao

The affiliation for the 18^th^ author is incorrect. The correct affiliation is not indicated. Annibale Cois is not affiliated with #3 and #12 but with: Burden of Disease Research Unit, South African Medical Research Council, Cape Town, South Africa.

## References

[1] Hussey H, Vreede H, Davies M-A, Heekes A, Kalk E, Hardie D, et al. SARS-CoV-2 seroepidemiology in Cape Town, South Africa, and implications for future outbreaks in low-income communities. PLOS Glob Public Health. 2024;4(8):e0003554. doi: 10.1371/journal.pgph.0003554 39106267 PMC11302865

