# Supplementary material for: SARS-CoV-2 seroepidemiology in Cape Town, South Africa, and implications for future outbreaks in low-income communities
Source: PLOS Glob Public Health. 2024 Aug 6;4(8):e0003554. doi: 10.1371/journal.pgph.0003554 (PMC11302865; doi:10.1371/journal.pgph.0003554)
Supplement: Attachment — Submitted filename: Response to reviewer_final.docx [file pgph.0003554.s001.docx]

**SARS-CoV-2 seroepidemiology in Cape Town, South Africa, and implications for future outbreaks in low-income communities**

Response to PLOS Global Health reviewer

January 2024

**Journal Requirements:**

*1. Please update your online Competing Interests statement. If you have no competing interests to declare, please state: “The authors have declared that no competing interests exist.”*

Online competing interests’ statement has been updated to declare that the authors have no competing interests now.

*2. Please provide separate figure files in .tif or .eps format only and ensure that all files are under our size limit of 10MB.*

Figures have been converted to .tif format now.

**Reviewer #1: Overall comments**

*This is a valuable paper describing seroprevalence and antibody positivity correlation with protection against severe disease in South Africa. I commend the authors for their research, which is also well situated in a global health context and providing insights on seroepidemiology for vulnerable populations.

My main major comment is to please situate context for the study for the population, sampling, and discussion where possible – i.e. describing the population demographics and landscape, vaccine availability and types available in the region over time, and any changes in testing policy over the course of the study, given the sampling date range is very broad. I would also suggest the authors consider bolstering their analyses with test and/or population adjustment for seroprevalence estimates. Please also add more comparison of your results with other literature measuring antibody positivity correlation with protection against severe disease in the discussion section.*

Thank you for this helpful suggestion. We agree with the reviewer that describing the context of the study is important and aids interpretation. In the Methods section, we describe the SARS-CoV-2 testing policy now (line 132): “With limited testing capacity in the Western Cape, SARS-CoV-2 testing was limited to those with severe disease, old age or medical comorbidities, particularly during the peaks of the waves, although a slight increase in testing capacity was noted from October 2020 when rapid antigen tests became available (19).”

In the Methods section we have also added more on the vaccination landscape in South Africa (line 158): “From 17 May 2021, primary vaccination with either BNT162b2 (2 doses) or Ad26.COV2.S (single dose) was available in South Africa, first to adults aged 60 years and above, and then progressively to younger age groups, with all individuals aged 12 years and older being eligible from 20 October 2021 (20).”

Sections have been added to the discussion now, that compare our seroprevalence findings to other studies, as well as that relate our findings on hybrid immunity (line 384): “A recent meta-analysis with studies from Europe and the Americas, found that while immunity wanes with time, individuals with this type of hybrid immunity had the highest magnitude and durability of protection against severe COVID-19, with protection of 97.4% at 12 months (95%CI 91.4-99.2), compared to 74.6% (95% CI 63.1-83.5) for previous infection alone after the same time period (29)”.

In the Results section, we have compared the demographics of our study sample to that of the general population in Cape Town (line 219): “Of the 16 636 specimens included, 68.5% were from females. 8.4% and 13.2% were from those aged 14 years and under and 65 years and older, respectively. This is in comparison to the 2022 Cape Town Census results (not fully released yet) which found that the population is 51.7% female; 22.4% were aged 14 and under and 6.7% aged 65 years and older (24). 37.1% of our samples were taken from diabetic patients and 41.6% were taken from people living with HIV. To note, the HIV prevalence in the Western Cape was estimated to be 10.3% in adults aged 15-49 years in 2018 (25), and the prevalence of diabetes in South African adults could be as high as 22% if undiagnosed diabetes is taken into account (25,26).”

We have also highlighted this again in the limitations section (line 407): “The residual samples we used also had a higher proportion of women and older adults, compared to the general population of Cape Town. While representativity of the general population is somewhat compromised by using residual samples, this study would not otherwise be feasible to perform in our setting, and we are still able to assess the differences in seroprevalence across various population groups.”

As our seroprevalence was assessed from residual specimens of largely diabetic or HIV positive patients, we have chosen to not adjust the study seroprevalence estimates by demographic characteristics, because even if we did that, our estimates will still be highly biased by the comorbidities of HIV or diabetes, due to the pragmatic nature of our study design with its convenience sampling of residual specimens.

Comments:

**Abstract**

*Line 24- Suggest deleting ‘determinants’ or rephrasing for clarity.*

Have now rephrased to “seroprevalence studies can help describe and characterise the extent of the pandemic.”

*Line 33-34: Please include 95% confidence intervals for results if available.*

Confidence intervals have been added.

*Could you include a short line on how you elucidated protection against severe disease and reported COVID-19 tests (what is your data source?)*

The text “Using COVID-19 hospital admission and death data at the Provincial Health Data Centre” has now been added.

**Introduction:**

*Line 60-63: How do these excess deaths compare to non-COVID excess deaths? Important to have a comparison to make your statement on possible case under-ascertainment stronger.*

No direct comparison is available, but we acknowledge that not all excess deaths are COVID related in the text: “While not all these deaths were directly due to SARS-CoV-2 infection, the fact that excess deaths were three times higher than reported deaths highlights the high levels of case under-ascertainment.”

*Line 63: Note typo with extra space and period.*

Typo corrected now.

*Line 65: What are the barriers to testing?*

The following text has now been added at line 66: “Barriers to testing are both at the individual level, including health literacy, trade-offs of knowing results and personal costs of time and out-of-pocket expenses to get tested, as well as at the health system level, with limited health system preparedness and laboratory capacity in poorer settings (4).”

*Line 70: Please elucidate elements needed to capture immune protection – i.e. quantitative antibody testing to detect antibody wane, etc. It is not just longitudinal versus cross sectional that limits these results being available. Also, for your consideration, here are a few South African serosurveys that are longitudinal, which you may wish situate the background in the context of these findings:*
[*https://www.mdpi.com/1999-4915/14/6/1222*](https://www.mdpi.com/1999-4915/14/6/1222)
[*https://www.mdpi.com/2077-0383/12/2/529*](https://www.mdpi.com/2077-0383/12/2/529)*.*

At line 89, the following text has been added: “While SARS-CoV-2 antibody positivity does wane with time (13), by assessing the sero-positivity between waves, and comparing that to the recorded infections in the preceding and subsequent waves, we are able to assess the impact of seropositivity beyond what a more traditional cross-sectional study would be able to do.”

In the Limitations section (line 406) we have also added: “antibody results were assessed as positive or negative only, and the quantitative antibody values, which would give an indication of waning protection with time, were not examined.”

The two references have been added to the Introduction (references 9 and 10), as well as the Discussion section.

**Methods:**

*Line 99: Could you please give an indication of approximately how many samples were processed by NHLS over the course of the study duration?*

At line 113, the following text has now been added: “In that time period, over 600 000 specimens were processed by the NHLS in the Cape Town Metro, including 193 993 HbA1c specimens and 412 539 HIV VL specimens.”

*Line 102: Please describe more information on the convenience sampling method used. I.e. did you select all samples on certain dates, first N of samples per month, etc, or were all samples included?*

No sample size calculation was used. Consecutive samples per sub-district per sampling interval were selected, until a predetermined number, subject to available budget, was met.

*Lines 101-107: Were there any exclusion criteria, or a sample size calculation used? If so, please describe.*

Please see the comment above. The only exclusion criteria we had were that if on visual inspection the specimens were grossly haemolysed or likely had insufficient volume.

The following text has been added at line 117: “Consecutive samples per sub-district per sampling interval were selected, until a predetermined number, subject to available budget, was met. Samples were excluded if on visual inspection they were found to be grossly haemolysed or likely to have insufficient volume.”

*Lines 110-111: Somewhere appropriate in the paper please describe the vaccination landscape in South Africa at the times of sampling, and which vaccines were predominantly administered (N-detecting assays for prior infection will not work if the population has been largely vaccinated with a non-mRNA vax such as the Oxford-Astra Zeneca vax).*

As mentioned above, the timelines of the introduction of the BNT162b2 and Ad26.COV2.S vaccines have been added to the Methods section.

Both the BNT162b2 and Ad26.COV2.S , which were the only vaccines available in South Africa, do not produce an anti-N response, as they are an mRNA and a vectored vaccine respectively, both based on viral spike sequences. The following text has been added at line 159: “Both of these vaccinations are based on viral spike sequences and therefore do not provoke an anti-N antibody response.”

*Line 112-115: Please describe the performance testing in South Africa if possible. It would also be advantageous to list the denominator for Sn and Sp testing (i.e., how many samples were used to determine the performance)?*

The assay was evaluated in several local and international studies and is widely used in published seroepidemiology studies. As such, beyond the existing citation, we don’t think detailed description of its performance is necessary. The cited study was a clinical validation consisted of 434 PCR tested participants’ samples that included negative samples (*n* = 124) and positive samples (*n* = 310).

Line 124-147: How was the vaccination status data analytically linked with the natural infection elucidation from your anti-N assay, particularly for Janssen, as recipients of a viral vector-based vaccine would produce anti-N antibodies?

For the natural infection elucidation, we linked the serology results to documented COVID-19 PCR or antigen tests at the Provincial Health Data Centre (PHDC) using the unique patient identifiers available there. As testing services in our setting were limited to those with symptomatic COVID-19 disease in high-risk population groups, like the elderly or those with comorbidities, it is unlikely that asymptomatic patients (who were only anti-N positive following vaccination) would receive a test. In any case, the Ad26.COV2.S vector-based vaccine only expresses spike and thus does not illicit an anti-N antibody response.

*Line 151-153: The sensitivity of the assay used in this paper is rather low. I would consider statistically adjusting the seroprevalence estimate by test performance.*

As this paper is focused on the trend of seroprevalence and the differences between various population groups, we have decided not to adjust for test performance. In addition, towards the end of the study period most samples were positive, so adjusting for the relatively low sensitivity of the assay would not substantially impact the overall findings.

*Line 167: Please provide specific date windows for each ‘wave’ of sampling.*

Dates are provided in the summary graphic (Figure 1), but have now also been added into the manuscript in line 230: “Anti-N seroprevalence increased from 39.19% (95% confidence interval [95%CI] 37.23-41.19%) in the first round (i.e., after the first wave, 17 August to 6 November 2020), to 50.12% (95%CI 48.60-51.64%) in the second round (8 February to 21 May 2021), and to 67.80% (95%CI 66.31-69.25%) in the third round before the start of the fourth wave (20 September to 27 November 2021).”

**Results**

*198: Replace epidemic with pandemic.*

Replaced now.

*Line 211-213: This sentence is confusing, consider re-phrasing or breaking up results into different sentences.*

Thank you for highlighting this issue. That sentence has now been broken up into three: “Higher seroprevalence was observed in individuals aged 17-49, compared to other age groups. Women had higher seroprevalence than men. People living with HIV also had higher seroprevalence than those with diabetes.”

**Discussion**

*Line 327: On this result on hybrid immunity – I would move this earlier to the top of your discussion with your other remarks on anti-N protection against severe disease, this is a higher-impact result. It would also be great to situate these results against VE or PE studies/meta-analyses – understanding this is not a VE or PE study in itself, but would be interesting to compare.*

While we agree that the findings around hybrid immunity are important, they are well established in the literature already, and the differential case ascertainment in poorer communities is the main novel finding of this study. We would therefore prefer not to restructure the Discussion section.

*Line 342-344: If you have demographic information available, you could statistically adjust for population representativeness.*

As mentioned earlier, while we could adjust for demographic characteristics, such as age and sex, we would not be able to adjust for the comorbidities of HIV and diabetes, as these were selection criteria for being included in the study, and so adjusting for population representativeness is not attainable within our current pragmatic study design that uses residual samples.
